# RITPBC: B-cell depleting therapy (rituximab) as a treatment for fatigue in primary biliary cirrhosis: study protocol for a randomised controlled trial

**DOI:** 10.1136/bmjopen-2015-007985

**Published:** 2015-08-20

**Authors:** Laura Jopson, Julia L Newton, Jeremy Palmer, Achilleas Floudas, John Isaacs, Jessica Qian, Jennifer Wilkinson, Mike Trenell, Andrew Blamire, Denise Howel, David E Jones

**Affiliations:** 1 Institute of Cellular Medicine, Medical School, Newcastle upon Tyne, UK; 2 Institute of Health and Ageing, Medical School, Newcastle upon Tyne, UK; 3 Newcastle Clinical Trials Unit, Institute of Health and Society, Newcastle University, Newcastle upon Tyne, UK; 4 Movelab @ Newcastle University, Medical School, Newcastle upon Tyne, UK; 5 Institute of Cellular Medicine, Newcastle Magnetic Resonance Centre, Newcastle University, Newcastle upon Tyne, UK; 6 Institute of Health and Society, Newcastle University, Newcastle upon Tyne, UK

**Keywords:** IMMUNOLOGY

## Abstract

**Introduction:**

Primary biliary cirrhosis (PBC) is an autoimmune liver disease with approximately 50% of patients experiencing fatigue. This can be a particularly debilitating symptom, affecting quality of life and resulting in social isolation. Fatigue is highlighted by patients as a priority for research and patient support groups were involved in designing this trial. This is the first randomised controlled trial to investigate a treatment for fatigue in PBC. The trial protocol is innovative as it utilises novel magnetic resonance spectroscopy (MRS) techniques as an outcome measure. The protocol will be valuable to research groups planning clinical trials targeting fatigue in PBC and also transferrable to other conditions associated with fatigue.

**Methods and analysis:**

RITPBC is a Medical Research Council (MRC) and National Institute for Health Research (NIHR) Efficacy and Mechanism Evaluation Programme (EME)-funded project. It is a phase II, single-centre, randomised controlled, double-blinded trial comparing rituximab with placebo in fatigued PBC patients. 78 patients with PBC and moderate to severe fatigue will be randomised to receive two infusions of rituximab or placebo. The study aims to assess whether rituximab improves fatigue in patients with PBC, the safety, and tolerability of rituximab in PBC and the sustainability of any beneficial actions. The primary outcome will be an improvement in fatigue domain score of the PBC-40, a disease-specific quality of life measure, evaluated at 12-week assessment. Secondary outcome measures include novel MRS techniques assessing muscle bioenergetic function, physical activity, anaerobic threshold and symptom, and quality of life measures. The trial started recruiting in October 2012 and recruitment is ongoing.

**Ethics and dissemination:**

The trial has ethical approval from the NRES Committee North East, has Clinical Trial Authorisation from MHRA and local R&D approval. Trial results will be communicated to participants, presented at national and international meetings and published in peer-reviewed journals.

**Trial registration number:**

ISRCTN03978701.

**Strengths and limitations of this study**

- RITPBC is the first randomised controlled trial of a treatment for fatigue in patients with primary biliary cirrhosis (PBC).
- The trial describes novel mechanistic outcome measures using magnetic resonance spectroscopy (MRS).
- Novel recruitment strategies utilising the UK-PBC trials platform are described.
- The main limitation is the responsivity of the primary outcome measure to meaningful improvement in fatigue. Responsivity is one of the 6 psychometric properties of any measure and determines its ability to measure meaningful change in a symptom in response to effective therapy. It is the untested one for the PBC-40 fatigue domain for the obvious reason that there is no therapy able to improve fatigue to allow us to test it. We have mitigated against this by having an objective measure as well (activity) and biomarkers (MRS).

## Background

Primary biliary cirrhosis (PBC) is a chronic cholestatic liver disease with a prevalence of 30/100 000.^1^ It affects approximately 20 000 people in the UK, predominantly females (10:1).^2^ PBC has an autoimmune aetiology with the majority of patients expressing autoantibodies directed against mitochondrial and nuclear antigens,^3^ and has strong associations with other autoimmune diseases^4^ PBC is characterised by inflammation and subsequent loss of the small intrahepatic bile ducts. Despite its name only a small number of patients will progress to cirrhosis and end-stage liver disease and a number of these will require liver transplantation.^5^ There is only one licensed treatment, ursodeoxycholic acid (UDCA), which slows progression of liver disease.^6^

PBC is associated with a number of symptoms including fatigue, cognitive impairment and pruritus. There are treatments available for PBC-associated pruritus but at present there are no licensed treatments for fatigue or cognitive dysfunction. Fatigue, which patients describe as physical exhaustion or their ‘batteries running down’, can be a very debilitating symptom. Fatigued patients are often unable to carry out normal day-to-day activities and frequently are unable to work resulting in a negative impact on quality of life.^7^ Recent data suggests that when patients have social isolation in combination with fatigue, there is a negative impact on quality of life.^8^ One patient with PBC has very eloquently described the impact of fatigue from PBC and the loneliness, frustration and despair that can result.^9^ In addition to the impact on the patients and their friends and family, fatigue is associated with economic costs (loss of earning, paying for assistance). Interestingly, fatigue is not related to the severity of liver disease^10^ and is unresponsive to UDCA therapy,^11^ suggesting that the processes responsible for fatigue in PBC are linked to the condition but not directly to liver injury.

There have been many advances in our understanding of fatigue over the years, starting initially with recognising it as a symptom associated with PBC, and then appreciating the scale of the problem^10^ and the impact it has on the patient's quality of life.^8^ Fatigue is a subjective symptom and not particular easy to study in clinical trials, and therefore the development and validation of the PBC-40, a disease-specific quality of life questionnaire, provides a key tool for studying fatigue in PBC.^12^ Studies have improved our understanding of the potential mechanisms driving fatigue in PBC and culminated in this clinical trial of rituximab. MRI studies point to an abnormality in muscle bioenergetic function as a potential cause of fatigue in PBC. Using the methods of phosphorous magnetic resonance spectroscopy (31P-MRS) they have demonstrated excessive intramuscular acidosis during and in the recovery from exercise. The severity of fatigue that patients describe is associated with the length of time that the acidosis is present and the recovery time back to the baseline pH.^13^ As a result of these observations, we hypothesise that the antimitochondrial antibodies which are seen in over 90% of people with PBC lead to over-utilisation of anaerobic pathways through their activity against pyruvate dehydrogenase (PDH). This is supported by MRS studies which have shown that mitochondrial dysfunction is directly related to antimitochondrial antibody (AMA) levels.^14^ Unpublished data from the Newcastle group have demonstrated significantly lower anaerobic threshold (AT) values compared with patients with age-matched and sex-matched primary sclerosing cholangitis (PSC) and sedentary controls, again supporting a muscle bioenergetic abnormality contributing to the fatigue seen in PBC. These findings support a trial to investigate rituximab, an anti-CD20, B-cell depleting monoclonal antibody as a potential treatment for fatigue in PBC.

To date there have been two pilot studies of rituximab in PBC.^15^ ^16^ Neither had fatigue as a primary outcome measure, and instead focused on UDCA-unresponsive disease and biochemical outcomes. Both studies found that rituximab resulted in an improvement in liver biochemistry and is safe and well tolerated in PBC. The pilot study in Canada reported a clinically significant reduction in fatigue but the trial was not optimised for the study of fatigue and did not include fatigue in the inclusion criteria, therefore potentially underestimating the clinical effect of rituximab on fatigue.^16^

The RITPBC trial aims to investigate rituximab as a treatment for fatigue in PBC. RITPBC will provide important data on the efficacy, safety and tolerability of rituximab in PBC, as the pilot studies have suggested there may be a role for rituximab in treating UDCA-unresponsive disease. The second and important aspect of the trial will be the mechanistic data which aims to gain a better understanding of the mechanisms underpinning fatigue in PBC, and if a benefit is seen, to establish the mechanism of action. The novel MRS techniques used as a secondary outcome measure are unique to this trial. Given that fatigue is not specific to PBC, any advances in the understanding of the mechanisms of this symptom are likely to be transferrable to other conditions associated with fatigue.

Research has been moving forward over the past 20 years to improve our understanding of the impact of fatigue and identify potential mechanisms but clinical trials of therapeutic agents have been lacking. Patients highlight fatigue as a priority for research and close working with the patient support groups re-iterates that trials and subsequent treatments for fatigue are much needed. RITPBC is the first randomised controlled trial of a therapeutic agent to target fatigue in PBC. RITPBC also pilots novel recruitment techniques through utilisation of the UK-PBC stratified medicine trials platform. If this recruitment strategy proves successful it has the potential to revolutionise trials in PBC and provide an approach for trial recruitment transferrable to any rare disease. This article discusses the protocol design and methodology and will hopefully aid further trials to target fatigue in PBC as well as being transferrable to the many other conditions that have fatigue as a prominent symptom.

## Methods/design

### Study design

RITPBC is a phase II, single-centre, randomised controlled, double-blinded trial comparing rituximab with placebo in fatigued PBC patients. A total of 78 patients (39/arm) with definite or probable PBC and moderate–severe fatigue (PBC-40 fatigue domain score >33) will be recruited. The primary outcome is improvement in fatigue severity, assessed using the PBC-40, a disease-specific quality of life measure,^12^ which will be evaluated at baseline and at 12 weeks. The secondary objectives will be to explore the extent to which any such improvement in fatigue is related to a reduction in the level of anti-PDH antibody and to any associated effect on biomarkers of bioenergetic function assessed using novel MRS protocols. Other secondary outcome measures are improvement in AT, physical activity levels, liver biochemistry, daytime somnolence, autonomic symptoms, functional status, cognitive dysfunction and anxiety and depressive symptoms. The safety of rituximab in PBC will be explored and the sustainability of any beneficial actions evaluated.

### Screening, recruitment and consent

Recruitment will principally be from the large clinical cohort under follow-up at the Joint Autoimmune Liver Disease Clinic in the Newcastle upon Tyne Hospitals NHS Foundation Trust (>500, the largest clinical PBC service in the UK). Participant Identification Centres (PICs) in hospital trusts in the North of England will be opened to aid recruitment. Articles will be written for the patient support group LIVErNORTH and PBC Foundation and published in their newsletters and magazines. This trial is unique as it was the first trial to utilise the UK-PBC platform for recruitment to trials. UK-PBC is a £5 million Medical Research Council (MRC)-stratified medicine trials platform which holds genetic, symptom and biochemical data on over 3000 patients with PBC in the UK. Patients recruited to UK-PBC have consented to be contacted about trials and can therefore be easily accessed and recruited. The UK-PBC database can be searched for patients with a PBC-40 fatigue domain score >33. The clinicians looking after these patients can then be contacted and can approach the patients to see if they would be interested in taking part in the trial. This recruitment strategy targets patients with moderate to severe fatigue on a national level that would be impossible to do in a single-centre trial.

Potentially eligible participants are given a study Participant Information Sheet (PIS) after which they will have a minimum of 48 h to consider this information before written informed consent is obtained.

Participants will have a diagnosis of definite or probable PBC established using recognised epidemiological criteria.^17^ ^18^

### Eligibility criteria

**Inclusion criteria**

Age ≥18 years

Patient has capacity and provided written informed consent for participation in the study prior to any study-specific procedures

Moderate or severe fatigue as assessed using previously designated cut-offs of the PBC-40 fatigue domain score (ie, fatigue domain score >33)

Presence of AMA at a titre of >1:40

Adequate haematological function haemoglobin >9 g/L, neutrophil count >1.5×10^9^/L, platelet count >50×10^9^/L

Bilirubin <50 µmol

International normalised ration ≤1.5

Child-Pugh score <7

Eastern Cooperative Oncology Group (ECOG) performance status <2

Adequate renal function; Cockcroft and Gault estimation >40 mL/min

Women with child-bearing potential should have a negative pregnancy test prior to study entry and be using an adequate contraceptive method which must be continued for 12 months after completion of treatment

**Exclusion criteria**

History or presence of other concomitant liver diseases (including hepatitis due to hepatitis B or C or evidence of chronic viraemia on baseline screening), primary sclerosing cholangitis or biopsy-proven non-alcoholic steatohepatitis

Average alcohol ingestion >21 units/week (male) or >14 units/week (female)

Chronic sepsis or intercurrent condition likely to predispose to chronic sepsis during the study

Previous history of aberrant response or intolerance to immunological agents

Presence of significant untreated intercurrent medical condition itself associated with fatigue

Presence of significant risk of depressive illness (Hospital and Anxiety and Depression Scale (HADS) score indicating caseness)

Current statin therapy or statin therapy within 3 months of enrolment

Ongoing participation in other clinical trials or exposure to any investigational agent 4 weeks prior to baseline or within <5 half-lives of the investigational drug

Major surgery within 4 weeks of study entry

Vaccination within 4 weeks of study entry; patients requiring seasonal flu or travel vaccines will be required to wait a minimum of 4 weeks postvaccination to enrol in the study

Pregnant or lactating women

Psychiatric or other disorder likely to impact on informed consent

The patient is unable and/or unwilling to comply with treatment and study instructions

Any other medical condition that, in the opinion of the investigator, would interfere with safe completion of the study

Hypersensitivity to the active substance (rituximab) or to any of the excipients (sodium citrate, polysorbate 80, sodium chloride, sodium hydroxide, hydrochloric acid, water (for injection)) or to murine proteins

Active, severe infections (eg, tuberculosis, sepsis or opportunistic infections)

Known HIV infection

Clinical history of latent tuberculosis infection unless the patient has completed adequate antibiotic prophylaxis

Alanine transaminase (ALT)/aspartate transaminase (AST) 4× upper limit of normal

Severe immunocompromised state

Severe heart failure (New York Heart Association (NYHA) Class IV) or severe uncontrolled cardiac disease

Malignancy (other than basal cell carcinoma) within the last 10 years

Demyelinating disease

Previous participation in this study

Any contraindication to rituximab therapy not covered by other exclusions

### Intervention

Patients in the study will be randomised on a 1:1 ratio to receive either:

- Rituximab 1 g on days 1 and 15—study drug
- 250 mL 0.9% sodium chloride on days 1 and 15—placebo.

### Experimental intervention: rituximab

The investigational medicinal product used in the clinical trial is rituximab, 1000 mg intravenously. This product has been approved with the European Commission decision (MA number: EU/1/98/067/002). Patients randomised to receive rituximab therapy will be given treatment at the infusion rates recommended for rheumatoid arthritis patients as per the Newcastle upon Tyne Hospitals NHS Foundation Trust protocol.

### Control intervention: placebo

Patients randomised to receive placebo will receive a control infusion of normal saline. The control infusion will be delivered in a double blinded manner to participants using the same protocol.

### Conditioning

In line with recommendations for the administration of rituximab in other conditions, all patients will receive a conditioning regimen prior to the infusions of study medication on days 1 and 15 to maintain double blinding. The conditioning regimen compromises paracetamol 1 g orally and chlorpheniramine 4 mg orally to be administered 1 h prior to infusion and methylprednisolone 100 mg intravenously to be administered 30 min prior to infusion.

### Concomitant medications

For patients who are on UDCA, the dose will not be changed during the period of study. No other disease-modifying agents should be introduced during the duration of the trial. Therapy aimed at reducing pruritus can be introduced if unavoidable at the discretion of the investigators.

Live vaccines must not be given during the study.

### Randomisation and blinding

Randomisation will be conducted by the Newcastle Clinical Trials Unit (NCTU) web-based system on a 1:1 ratio and random-permuted blocks with random block length. The treatment allocation will be kept blind from the patients, study assessors and investigators until study completion. The randomisation system will generate a treatment number for each participant that links to the corresponding allocated study drug (blinded). A code-break list will be kept in the pharmacy department.

### Study procedures and outcome measures

**Figure 1:**
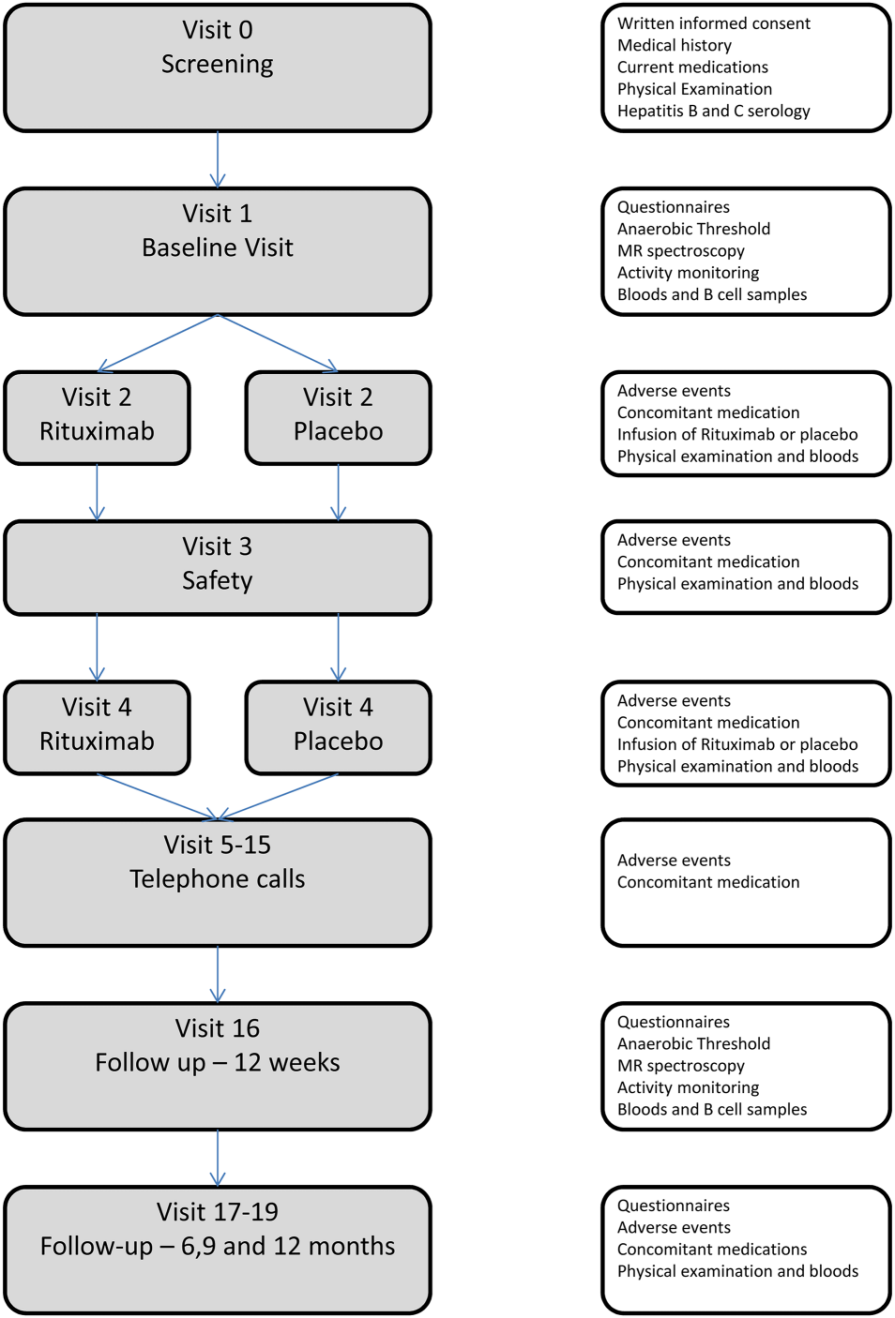
Schedule of events for the RITPBC trial.

The schedule of events which includes completion of study questionnaires and collection of mechanistic data is described in figure 1.

### Quality of life and symptom questionnaires

The primary outcome measure is the fatigue domain of the PBC-40. Severity of other symptoms will be assessed in terms of numerical change for the relevant domain of the PBC-40 (itch, cognitive symptoms, social and emotional symptoms). The PROMIS HAQ (Patient-Reported Outcomes Measurement Information System Health Assessment, Questionnaire)^19^ measures the functional and physical ability of the participants (washing, dressing, arising, eating, walking, hygiene, reach, grip and activity). The score is on a 0–100 scale with higher scores indicating worse functional ability. Anxiety and depression will be assessed by the HADS.^20^ Improvement in daytime somnolence will be assessed using the Epworth Sleepiness Scale (ESS).^21^ Vasomotor autonomic symptoms will be assessed using the Orthostatic Grading Scale (OGS).^22^Cognitive function will be assessed with the COGFAIL questionnaire.^23^

### Fatigue diary

Participant-held diaries will be used to gather qualitative information as to symptoms and functional ability. We will use diaries that include both structured (quantitative) and unstructured (qualitative) methods of data collection. The diaries measure fatigue using a scale of 1 to 6 (1—no fatigue and 6—extreme fatigue). Participants will be asked to complete the diaries six times during the study. They will complete the diaries for a period of 1 week at the beginning of each month at baseline, 1, 3, 6, 9 and 12 months. They will return the diaries at the final visit.

### Muscle acidosis

Magnetic resonance data will be acquired at baseline and at 12 weeks using a 3 T Intera Achieva scanner (Philips, Best, the Netherlands) which is equipped with additional specialist hardware to perform phosphorous-31 MRS. The protocol used for acquisition and analysis has been described elsewhere; but in summary, it involves controlled plantar flexion using a purpose-built exercise apparatus developed for operation within the MRI scanner.^14^ Participants will perform 2×180 s bouts of plantar flexion contractions at 25% and then 35% of maximal voluntary contraction, with each bout preceded by 60 s of rest and followed by 390 s of recovery. Phosphorous spectra will be collected at 10 s intervals.

### Anaerobic threshold

Participants will cycle on a stationary ergometer (Corival, Lode, Nederland) between 60 and 70 rpm. The test will be terminated voluntarily by the participant or when they were unable to maintain a pedal frequency of 60 rpm. Expired air will be collected at rest and during exercise using a breathing mask and analysed online using a gas analysis system (MetaLyzer II, CORTEX, Germany). AT will be assessed using the computerised v-slope method and values compared at baseline and 12-week follow-up.

### Physical activity levels

Physical activity will be measured objectively using two activity monitors which will be worn for 7 days at baseline and week 12. The first is a validated multisensor array (SenseWear Pro_3_, Bodymedia Inc) which measures four key metrics: skin temperature, galvanic skin response, heat flux and motion via a three-axis accelerometer. The sensors combined with algorithms calculate the average daily energy expenditure relative to baseline metabolism (metabolic equivalent: MET/day, 1 MET=resting metabolic rate), total energy expenditure (calories per day), active energy expenditure (total calories expended over 3 MET/day), physical activity duration (min over 3METs per day) and average daily number of steps walked. Patterns of sedentary behaviour will be assessed by power law analyses of the lengths of sedentary bouts fitted from raw sedentary data. Outcome measure for study evaluation will be mean number of steps/24 h. Second, the GENEActiv (ActivInsights, Ltd) is a waterproof, lightweight (16 g) triaxial accelerometer. Raw accelerations are collected at a range of ± 8 g with a recording frequency of 40 Hz.

### B-cell biology

Quantification and phenotyping of total B-cell populations and B-cell subsets will be carried out using a FACS-based approach with a well-described protocol utilising markers other than CD20.^24^ Outcome measure will be a chang in individual parameters with therapy. Total and activated B cells in peripheral blood will be evaluated using a direct immune-fluorescence reagent (Fast Immune CD19/CD69/CD45, BD Biosciences). B-cell subsets will be analysed to assess naïve, memory (CD27) and plasma cell (CD38) populations. Finally, activation status of the B-cell populations will be analysed using CD80, CD86 and CD268.

Anti-PDH antibody total and individual isotype levels and antibody functional inhibitory capacity will be studied on day 0 and at the primary end point (12 weeks after therapy). Antibody levels will also be correlated with long-term fatigue status during the secondary follow-up period to 12 months. Anti-PDH levels and isotype patterns will be assessed using a well-established ELISA developed within our research group.^25^ In the analysis phase, impact of rituximab on fatigue in PBC will be correlated with changes in individual autoantibody isotype responses and with PDH-inhibitory capacity of serum.

### Liver biochemistry

We will collect data on the reduction in serum alkaline phosphatase level and attainment of the previously identified positive outcome measure of drop in baseline alkaline phosphatase of >40% or normalisation (Barcelona Criteria).^26^

### Data analysis

#### Sample size calculation

A total sample size of 78 participants (39 per arm) will be recruited and randomised; this includes an assumption of 10% attrition at 12-week follow-up (based on experience in clinical trials in PBC). The primary outcome is the PBC-40 fatigue domain score (range 11–55) after 12 weeks of intervention. The SD of fatigue scores is 8 units (based on the PBC-40 derivation studies utilising >1000 patients^12^), with a correlation of 0.6 between baseline and follow-up time points based on previous studies. The study is powered to detect a mean change in PBC-40 fatigue domain score of 5 units (equating to an average of 0.5 point change per question; a difference in PBC-40 score demonstrated in our population-based studies to be associated with significantly higher levels of social function and which was, therefore, deemed to be clinically significant for the purposes of the study design) with a power of 90% and a 5% significance level. This equates to 35 participants in each group providing data on the primary outcome (PBC-40 fatigue score at 12 weeks): incorporating an assumption of a 10% attrition gives a total of 78. The number of participants lost to follow-up, or withdrawing consent prior to initial treatment is expected to be minimal.

#### Analysis of outcome measures

Analysis will be on the basis of intention to treat.

#### Primary outcome

Differences between intervention and control groups at 12 weeks on PBC-40 fatigue domain scores will be analysed by analysis of covariance (ANCOVA) using baseline scores as covariates. The time course of the comparison between intervention and control groups over the 12-month follow-up period will be assessed using repeated measures analysis of variance.

#### Secondary outcome

Secondary outcomes, covering comparison of other clinical symptoms and functional capability scales at 12 weeks will also be analysed by ANCOVA using baseline values as covariates. The time course of the comparison between intervention and control groups over the 12-month follow-up period will be assessed using repeated measures analysis of variance.

The analysis of the mechanistic variables will be more descriptive in nature, and will involve comparison of means or proportions between intervention groups, as appropriate, and the use of correlation coefficients to explore the relationship between physiological/immunological measurements and fatigue. For the secondary outcome measures, the variables will be compared at baseline and at 12-week assessment. Physical activity levels will compare mean number of steps per 24 h measured over a 7-day period. AT will be measured during cardiopulmonary exercise testing. MRS will compare the minimum pH seen in the exercise and recovery period, the time required postexercise for pH to return to within 0.01 units of baseline levels (calculated as the sum for each individual of the two bouts to form a total pH recovery time) and the mean ‘area under the curve’ for pH for the 2 exercise episodes which reflects total acid exposure. A change in the number of B cells and B-cell subsets will be reported and also a change in anti-PDH titres. The safety and tolerability of rituximab in PBC will also be reported as a secondary outcome measure.

There are no planned interim analyses for efficacy; however, if the data monitoring and ethics committee (DMEC) requires interim analysis for safety it will be performed. Final analyses will be carried out when all participants have completed the follow-up.

### Withdrawal of participants

Study drug must be discontinued if:

- The participant develops elevated serum ALT/AST four times above the upper limit of normal;
- The participant decides she/he no longer wishes to continue;
- Cessation of study drug is recommended by the investigator.

Should a patient withdraw from study drug only, efforts will be made to continue to obtain follow-up data, with the permission of the patient.

Participants who wish to withdraw from study medication will be asked to confirm whether they are still willing to provide the following:

- Study specific data at follow-up visits 5–19;
- End of study data as per visit 19, at the point of withdrawal;
- Questionnaire data collected as per routine clinical practice at annual follow-up visits.

If participants agree to any of the above, they will be asked to complete a confirmation of withdrawal form to document their decision.

### Data monitoring, quality control and quality assurance

#### Data collection

To preserve confidentiality, all patients will be allocated a unique study identification number, which will be used on all data collection forms and questionnaires. Only a limited number of members of the research team will be able to link this identification number to identifiable details, which will be held on a password-protected database. All study documentation will be held in secure offices and the research team will operate to a signed code of confidentiality. A clinical data management software package will be used for data entry and processing, allowing a full-audit trail of any alterations made to the data post entry. Original questionnaires, case report forms and consent forms will be securely archived at the Newcastle upon Tyne Hospitals NHS Foundation Trust archive facility for 15 years following publication of the last paper or report from the study. Data will be handled, computerised and stored in accordance with the Data Protection Act 1998. No participant identifiable data will leave the study site. The quality and retention of study data will be the responsibility of the chief investigator. All study data will be retained in accordance with the latest Directive on GCP (2005/28/EC) and local policy. All laboratory samples will be stored and identified using the patient's unique study identification code.

#### Discontinuation rules

The study may be prematurely discontinued on the basis of new safety information, or for other reasons given by the DMEC and/or Trial Steering Committee (TSC), Sponsor, regulatory authority or Research Ethics Committee concerned.

#### Monitoring, quality control and quality assurance

The study will be managed through the NCTU. The Trial Management Group (TMG) will include the chief investigator, senior trial manager, trial manager, assistant trial manager, data manager and other members of the trial team when applicable. NCTU will provide day-to-day support for the site and provide training through investigator meetings, site initiation visit and routine monitoring visits. Protocol amendments will be managed by the NCTU and communicated to the trial team.

#### Data Monitoring and Ethics Committee

An independent DMEC has been appointed. It will consist of two physicians not connected to the study and one independent statistician and will be convened to undertake independent review. The purpose of this committee will be to monitor efficacy and safety end points. Only the DMEC will have access to unblinded study data. The committee will meet a minimum of three times, at the start, middle and completion of the study.

#### Trial Steering Committee

A TSC will be established to provide overall supervision of the study. The TSC will consist of an independent chair, two independent clinicians, independent consumer representative, the chief investigator, co-investigator, senior trial manager, trial manager and trial statistician. Representatives of the trial sponsor and funder should be invited to all TSC meetings. The committee will meet every 3 months during recruitment, and annually thereafter for the duration of the study.

#### Study monitoring

Monitoring of study conduct and data collected will be performed by a combination of central review and site monitoring visits to ensure the study is conducted in accordance with good clinical practice (GCP). Study site monitoring will be undertaken by the trial manager. The main areas of focus will include consent, serious adverse events (SAEs), essential documents in study site files and drug accountability and management. All monitoring findings will be reported and followed up with the appropriate persons in a timely manner. The study may be subject to inspection and audit by the Newcastle upon Tyne Hospitals NHS Foundation Trust under their remit as sponsor, and other regulatory bodies to ensure adherence to GCP.

### Recording and reporting serious adverse events or reactions

All adverse events (AEs) should be reported. Depending on the nature of the event, the reporting procedures below should be followed. Any questions concerning AE reporting should be directed to the chief investigator or the named contact within the management team within the NCTU in the first instance.

#### Adverse event (including adverse reaction)

All non-SAEs/reactions during drug treatment will be reported on the study case report form (CRF) and sent to the NCTU management team within 2 weeks. Severity of AEs will be graded on a five-point scale (Mild, Moderate, Severe, Life threatening, causing death). Relation of the AE to the treatment should be assessed by the investigator at site. The individual investigator at each site will be responsible for managing all AEs/reactions according to local protocols.

#### SAE/SAR (including SUSARs)

All SAEs, SARs and SUSARs during drug treatment shall be reported to the chief investigator within 24 h of the site learning of its occurrence. The initial report can be made by secure fax which will also generate an email copy to the chief investigator, senior trial manager and trial manager. In the case of incomplete information at the time of initial reporting, all appropriate information should be provided as follow-up, on the appropriate SAE follow-up form. As indicated above, relationship of the SAE to the treatment (causality) should be assessed by the investigator at the site, as should the expected or unexpected nature (by reference to the SmPC for rituximab) of any serious adverse reactions (SARs). The MHRA and main REC will be notified by the chief investigator or trials management team (on behalf of the Sponsor) of all SUSARs occurring during the study according to the following timelines: fatal and life-threatening within 7 days of notification and non-life-threatening within 15 days. All investigators will be informed of all SUSARs occurring throughout the study on a case-by-case basis. The chief investigator will ensure the Newcastle upon Tyne Hospitals NHS Foundation Trust as Sponsor is notified of any SUSARs in accordance with local trust policy.

#### Insurance and finance

The Newcastle upon Tyne Hospitals NHS Foundation Trust has liability for clinical negligence that harms individuals towards whom they have a duty of care. National Health Service (NHS) Indemnity covers NHS staff and medical academic staff with honorary contracts conducting the study for potential liability in respect of negligent harm arising from the conduct of the study. The Newcastle upon Tyne Hospitals Foundation NHS Trust is sponsor and through the sponsor, NHS indemnity is provided in respect of potential liability and negligent harm arising from study management. Indemnity in respect of potential liability arising from negligent harm related to study design is provided by NHS schemes for those protocol authors who have substantive contracts of employment with the NHS and by Newcastle University Insurance schemes for those protocol authors who have substantive contract of employment with Newcastle University. This is a non-commercial study and there are no arrangements for non-negligent compensation.

## Dissemination

The data will be the property of the chief investigator and co-investigator(s). Publication will be the responsibility of the chief investigator. It is planned to publish this study in peer-review journals and to present data at national and international meetings. Results of the study will also be reported to the sponsor and funder, and will be available on their web site. All manuscripts, abstracts or other modes of presentation will be reviewed by the TSC and funder prior to submission. Individuals will not be identified from any study report. Participants will be informed about their treatment and their contribution to the study at the end of the study, including a lay summary of the results.

## Discussion

Over recent years, there have been many advances in gaining a better understanding of fatigue in PBC. We now appreciate what a significant problem it is, both in terms of the prevalence of fatigue as a symptom as well as the impact that it can have on the patient's lives. Although the pathophysiology of fatigue is not entirely understood research has enabled us to have a much better understanding of the potential causes of fatigue in PBC and possible targets for treatment. Given the recent increases in our understanding of fatigue the next step was to design and deliver a trial of a therapeutic agent aimed to improve fatigue in PBC which would also improve our understanding of the mechanisms of fatigue.

The trial is designed to provide useful information about the physiological effects of fatigue, the pathophysiology of fatigue as well as assessing the role of rituximab in treating fatigue.

Fatigue is a subjective symptom and is not always an easy symptom to study in a trial, which is why the protocol of this trial has been published with the hope of aiding others who may wish to design and deliver trials on fatigue in PBC as well as the many other conditions that have fatigue as a key symptom. The trial also incorporates novel techniques that can provide objective data on fatigue (MRS, AT and physical activity monitoring) as well as describing new recruitment techniques using national disease-specific databases. Forty patients have now received either rituximab or placebo infusions as part of the trial which make it the largest biological trial in PBC. We believe the high number of patients recruited to this trial is because we are addressing fatigue which is the issue that matters most to patients.

## Trial status

Recruitment to RITPBC opened in October 2012 and will close in April 2015.

At the time of manuscript submission the trial is open to recruitment.
